# Glucagon Stimulates Hepatic FGF21 Secretion through a PKA- and EPAC-Dependent Posttranscriptional Mechanism

**DOI:** 10.1371/journal.pone.0094996

**Published:** 2014-04-14

**Authors:** Holly A. Cyphert, Kimberly M. Alonge, Siri M. Ippagunta, F. Bradley Hillgartner

**Affiliations:** Department of Biochemistry, West Virginia University, Morgantown, West Virginia, United States of America; University of Toronto, Canada

## Abstract

Previous studies have shown that whole body deletion of the glucagon receptor suppresses the ability of starvation to increase hepatic fibroblast growth factor 21 (FGF21) expression and plasma FGF21 concentration. Here, we investigate the mechanism by which glucagon receptor activation increases hepatic FGF21 production. Incubating primary rat hepatocyte cultures with glucagon, dibutyryl cAMP or forskolin stimulated a 3-4-fold increase in FGF21 secretion. The effect of these agents on FGF21 secretion was not associated with an increase in FGF21 mRNA abundance. Glucagon induction of FGF21 secretion was additive with the stimulatory effect of a PPARα activator (GW7647) on FGF21 secretion. Inhibition of protein kinase A (PKA) and downstream components of the PKA pathway [i.e. AMP-activated protein kinase and p38 MAPK] suppressed glucagon activation of FGF21 secretion. Incubating hepatocytes with an exchange protein directly activated by cAMP (EPAC)-selective cAMP analog [i.e. 8-(4-chlorophenylthio)-2'-O-methyladenosine-3', 5'-cyclic monophosphate (cpTOME)], stimulated a 3.9-fold increase FGF21 secretion, whereas inhibition of the EPAC effector, Rap1, suppressed glucagon activation of FGF21 secretion. Treatment of hepatocytes with insulin also increased FGF21 secretion. In contrast to glucagon, insulin activation of FGF21 secretion was associated with an increase in FGF21 mRNA abundance. Glucagon synergistically interacted with insulin to stimulate a further increase in FGF21 secretion and FGF21 mRNA abundance. These results demonstrate that glucagon increases hepatic FGF21 secretion via a posttranscriptional mechanism and provide evidence that both the PKA branch and EPAC branch of the cAMP pathway play a role in mediating this effect. These results also identify a novel synergistic interaction between glucagon and insulin in the regulation of FGF21 secretion and FGF21 mRNA abundance. We propose that this insulin/glucagon synergism plays a role in mediating the elevation in FGF21 production during starvation and conditions related to metabolic syndrome.

## Introduction

Fibroblast growth factor 21 (FGF21) is a newly identified hormone that plays a role in mediating adaptive changes in carbohydrate and lipid metabolism in response to nutritional stress [1], [2]. For example, starvation causes a robust increase in FGF21 expression in liver, the predominant site of FGF21 production [3], [4], [5]. Elevated FGF21 levels play a role in mediating the increase in hepatic gluconeogenesis, fatty acid oxidation, and ketogenesis caused by starvation [3], [4], [6], [7]. FGF21 also increases plasma glucocorticoid concentration, modulates circadian behavior, and inhibits bone growth, growth hormone sensitivity, and female fertility [8], [9], [10], [11]. All of these effects occur during starvation, providing further evidence that FGF21 is a key mediator of the adaptive starvation response.

FGF21 has also drawn attention as a promising new approach to treat metabolic syndrome. Pharmacological administration of recombinant FGF21 increases energy expenditure, glucose tolerance, and insulin sensitivity and decreases adiposity, hyperlipidemia, and hepatic triacylglycerol accumulation in rodent and non-human primate models of obesity and type 2 diabetes [12], [13], [14], [15], [16]. Recent studies have shown that administration of a FGF21 analog is effective in lowering blood lipid and glucose levels in obese/type 2 diabetic human subjects [17]. One factor that has limited the development of exogenous FGF21 as a drug to treat metabolic syndrome is its short half-life in the circulation [18]. An alternative approach to treat metabolic syndrome is to develop drugs or nutritional supplements that induce a sustained increase endogenous FGF21 production. Accordingly, there is a strong interest in characterizing the physiological and molecular mechanisms controlling FGF21 production.

The mechanisms mediating the stimulatory effect of starvation on FGF21 production have been partially characterized. Activation of PPARα stimulates an increase in hepatic FGF21 gene transcription, and deletion of the PPARα gene suppresses the ability of starvation to induce FGF21 mRNA abundance [3], [4]. These observations have led to the proposal that alterations in PPARα activity play a role in mediating the starvation-induced increase in FGF21 gene expression.

Deletion of the PPARα gene does not completely inhibit the stimulatory effect of starvation on FGF21 mRNA abundance and plasma FGF21 levels suggesting that a PPARα-independent pathway(s) plays a role in the regulation of FGF21 expression [3], [4]. One possible pathway may be activated by the pancreatic hormone, glucagon. Starvation increases the secretion and plasma concentration of glucagon [19]. Glucagon binds to a transmembrane spanning G protein-coupled receptor that activates adenylyl cyclase resulting in an increase in cAMP production. cAMP activates signaling pathways that cause an increase in gluconeogenesis, glycogenolysis, and fatty acid oxidation and a decrease triacylglycerol synthesis and very low density lipoprotein production [19], [20], [21]. Recent studies have shown that whole body deletion of the glucagon receptor suppresses the ability of starvation to increase hepatic FGF21 mRNA abundance [22]. Thus, glucagon appears to play a role in mediating the effect of starvation on FGF21 gene expression.

The mechanism by which glucagon regulates hepatic FGF21 expression is unclear. Glucagon receptors are expressed in liver, kidney, adipose tissue, heart, intestinal smooth muscle, brain, and endocrine pancreas with the liver having the highest level of expression [19]. Glucagon may act directly on the liver to increase hepatic FGF21 expression. Such a mechanism has been described for the glucagon induction of the gluconeogenic enzymes, phosphoenolpyruvate carboxykinase (PEPCK) and glucose-6-phosphatase (G6PC) [23], the transcriptional coactivator, peroxisome proliferator-activated receptor-g coactivator-1α (PGC-1α) [24], the cell signaling antagonist, suppressor of cytokine signaling-3 (SOCS3) [25], Ca^+2^ and Cl^−^ channel activity [26], and hepatocyte survival [27], [28]. Glucagon regulation of PEPCK, G6PC, and PGC-1α expression is mediated by the cAMP activation of protein kinase A (PKA), resulting in the phosphorylation and activation of cAMP response element binding protein, a transcription factor that activates the transcription of the genes encoding PEPCK, G6PC, and PGC-1α [29], [30]. In contrast, glucagon regulation of SOCS3 expression, Ca^+2^ and Cl^−^ channel activity, and hepatocyte survival is mediated by a PKA-independent mechanism involving the cAMP activation of exchange protein directly activated by cAMP (EPAC), a guanine nucleotide exchange factor that activates the small GTPase Rap1 [25], [26], [27], [28], [31].

Glucagon may also act at an extrahepatic site to induce hepatic FGF21 expression. In this scenario, glucagon would alter the extrahepatic production of a hormone or metabolite that modulates hepatic FGF21 expression. For example, glucagon causes an increase in adipose tissue lipolysis in rodents and humans resulting in the increased delivery of non-esterified fatty acids to the liver [32], [33]. Long-chain unsaturated fatty acids have been shown to increase hepatic FGF21 mRNA abundance [3], [34]. Thus, glucagon could stimulate hepatic FGF21 expression by modulating the delivery of extrahepatic fatty acids to the liver.

In the present study, we investigated the role of the direct mechanism in mediating glucagon regulation of FGF21 expression by determining the effects of glucagon on FGF21 gene expression and FGF21 secretion in primary cultures of rat hepatocytes. We show that glucagon stimulates FGF21 secretion via a translational and/or posttranslational mechanism and that both the PKA branch and the EPAC branch of the cAMP pathway mediate this effect. We also report that insulin stimulates FGF21 secretion via a pretranslational mechanism and that glucagon synergistically interacts with insulin to induce a further elevation in FGF21 secretion and FGF21 expression. The significance of this insulin/glucagon synergistic interaction in the regulation of FGF21 production is discussed.

## Materials and Methods

### Cell culture

Hepatocytes were isolated from 24 h starved male Sprague Dawley rats (approximately 200 g) as described by Stabile et al. [35]. Cells (3×10^6^) were plated on 60 mm collagen-coated dishes containing Waymouth's medium MD752/1 supplemented with 20 mM HEPES, pH 7.4, 0.5 mM serine, 0.5 mM alanine, penicillin (100 mg/ml), streptomycin (100 mg/ml), gentamicin (50 mg/ml) and 5% newborn calf serum. At 4 h of incubation, the medium was replaced with one of the same composition lacking newborn calf serum. A Matrigel overlay (0.3 mg/ml) and insulin (50 nM) were added at this time. At 24 h of incubation, the cells were washed in serum-free Medium 199 lacking insulin, and the incubation was continued in serum-free Medium 199. At 48 h of incubation, the medium was replaced with one of the same composition. At 66 h of incubation, the medium was replaced with one containing the treatments indicated in the figure legends. Hepatocyte cultures were maintained in a humidified chamber at 37°C in 5% CO_2_/95% air. This study was carried out in strict accordance with the recommendations in the Guide for the Care and Use of Laboratory Animals of the National Institutes of Health. In preparing rat hepatocytes, all surgeries were performed under isoflurane anesthesia, and all efforts were made to minimize suffering. We would like to confirm that the West Virginia University Institutional Animal Care and Use Committee approved this study (Protocol Approval Number: 12-0804).

Human HepG2 cells and rat H4IIE cells were plated on 60 mm dishes containing DMEM supplemented with penicillin (100 mg/ml), streptomycin (100 mg/ml), and 10% FBS. After the cells reached 80% confluence, the medium was changed to one of the same composition lacking FBS. After 24 h of incubation, the medium was replaced with serum free-DMEM containing the treatments indicated in the figure legends.

Glucagon, dibutyryl cAMP, and GW7647 were obtained from Sigma-Aldrich. Forskolin and SB203580 were purchased from LC Laboratories. Bovine insulin was a gift from Lilly. Compound C (EMD Millipore), H89 (Cayman Chemical), and 8-(4-chlorophenylthio)-2'-O-methyladenosine-3', 5'-cyclic monophosphate (R&D Systems) were obtained from the indicated sources.

### Isolation of RNA and quantitation of mRNA levels

Total RNA was extracted from tissues and cell cultures by the guanidinium thiocyanate/phenol/chloroform method [36]. The abundance of mRNA encoding FGF21, PEPCK, and SOCS3 was measured by quantitative real-time PCR analysis using the QIAGEN Quantitect SYBR green RT-PCR system. Samples of DNase I-treated RNA (100 ng) were analyzed in triplicate according to the manufacturer's instructions. PCR was performed in ninety-six well plates using a Bio-Rad iCycler iQ. The relative amount of mRNA was calculated using the comparative C_t_ method. Rat cyclophilin and human glyceraldehyde 3-phosphate dehydrogenase were used as reference genes. Amplification of specific transcripts was confirmed by analyzing the melting curve profile performed at the end of each run and by determining the size of the PCR products using agarose electrophoresis and ethidium bromide staining. The sequences of the primer sets can be obtained from the corresponding author upon request.

### Measurement of the concentration of FGF21 and albumin in the culture medium

The concentration of FGF21 in the culture medium of primary rat hepatocytes, rat H4IIE cells, and human HepG2 cells was determined using a rat or human FGF21 ELISA (R & D Systems). The concentration of albumin in the culture medium was determined using a rat or human albumin ELISA (Aviscera Bioscience).

### Western analyses

Total cell extracts were prepared from hepatocytes as described by Hansmannel et al. [37] except that the lysis buffer contained 25 mM Tris-HCl, pH 7.6, 150 mM NaCl, 1% NP-40, 1% sodium deoxycholate, 0.1% SDS and a mixture of protease inhibitors and phosphatase inhibitors (Halt, Thermo Scientific). Equal amounts of denatured protein were subjected to electrophoresis in SDS-polyacrylamide gels and then transferred to polyvinylidene difluoride membranes (Immobilon-FL, Millipore) using an electroblotting apparatus (Bio-Rad). The blots were blocked in TBST (10 mM Tris-HCl, pH 8.0, 150 mM NaCl, and 0.1% Tween) containing 5% nonfat dry milk for 1 h at room temperature and then incubated with primary antibody diluted 1∶500 in TBST containing 5% bovine serum albumin. After incubation with primary antibody for 12 h at 4°C, the blots were washed in TBST. Next, the blots were incubated with secondary antibody conjugated to horseradish peroxidase (Jackson ImmunoResearch) diluted 1∶2000 in TBST, 5% nonfat dry milk for 1 h at room temperature. After washing with TBST, antibody/protein complexes on blots were detected using enhanced chemiluminescence (Amersham Biosciences). Fluorescence on the blots was visualized using a Typhoon 9410 imager and signals were quantified using ImageQuant software. Antibodies against phosphorylated acetyl-CoA carboxylase 1 (ACC1) Ser^79^/acetyl-CoA carboxylase 2 (ACC2) Ser^212^, phosphorylated p38 MAPK Thr^180^/Tyr^182^, total ACC1/ACC2, total p38 MAPK were obtained from Cell Signaling Technology.

### Transduction of rat hepatocytes with adenovirus

Rap1GAP and LacZ control adenoviruses were kindly provided by Dr. Alan Smrcka, University of Rochester. Large-scale production of adenovirus was carried out in Ad-293 cells. Virus was purified using the ViraBind Adenovirus Purification kit (Cell Biolabs) and viral titer was determined using the Adeno-X Rapid Titer kit (Clontech). Adenovirus constructs (200 viral particles per cell) were incubated with primary rat hepatocytes at 4 h of incubation. Subsequent experimentation was performed after 24 h of infection.

### Statistical methods

Data were subjected to analysis of variance and statistical comparisons were made with the Dunnett's or Student's *t*-test.

## Results

### Activation of the glucagon/cAMP pathway increases hepatic FGF21 secretion

**Figure 1:**
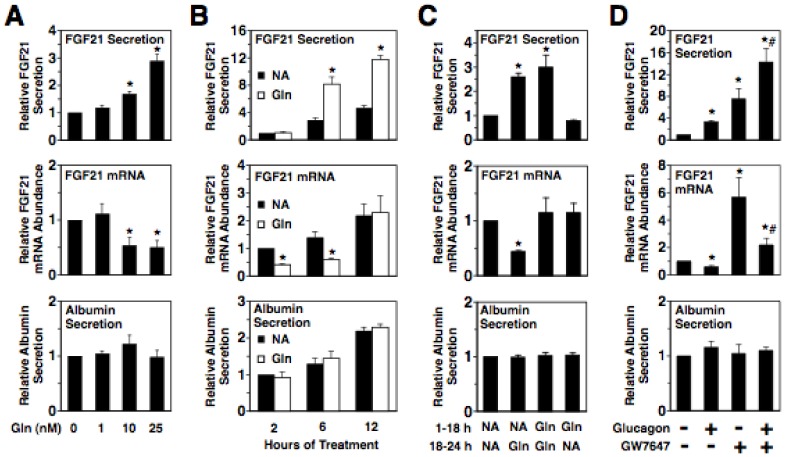
Glucagon increases FGF21 secretion via a mechanism not involving changes in FGF21 mRNA abundance. A: effect of different concentrations of glucagon on FGF21 secretion, FGF21 mRNA abundance, and albumin secretion in primary rat hepatocyte cultures. Cells were incubated with the indicated concentrations of glucagon in serum-free Medium 199 for 6 h. The level of FGF21 and albumin in the culture medium and the abundance of FGF21 mRNA in total RNA of cells incubated with 0 nM glucagon (Gln) were set at 1, and the other values were adjusted proportionately. Values are means ± SE (n = 3). B: time course of the effect glucagon on FGF21 secretion, FGF21 mRNA abundance, and albumin secretion. Rat hepatocytes were incubated with or without glucagon (25 nM) for the indicated time periods. The level of FGF21 and albumin in the culture medium and the abundance of FGF21 mRNA in total RNA of cells incubated with no additions (NA) for 2 h were set at 1, and the other values were adjusted proportionately. Values are means ± SE (n = 4). C: the effect of glucagon on FGF21 secretion is reversible. Rat hepatocytes were initially incubated with or without glucagon for 18 h. The culture medium was then replaced with one containing glucagon or NA, and the incubation was continued for 6 h. The level of FGF21 and albumin in the culture medium and the abundance of FGF21 mRNA in total RNA of cells incubated with NA for both treatment periods were set at 1, and the other values were adjusted proportionately. Values are means ± SE (n = 4). D: interaction between glucagon and PPARα in the regulation of FGF21 secretion and FGF21 mRNA abundance. Rat hepatocytes were incubated with or without glucagon (25 nM), GW7647 (1 µM), or glucagon plus GW7647 for 6 h. The level of FGF21 and albumin in the culture medium and the abundance of FGF21 mRNA in total RNA of cells incubated no additions were set to 1, and the other values were adjusted proportionately. Values are means ± SE (n = 3). * Significantly different (*P*<0.05) from that of cells incubated with no additions. ^#^ Significantly different (*P*<0.05) from that of cells incubated with glucagon or GW7647 alone.

Previous studies have shown that starvation increases plasma glucagon concentration and that whole body deletion of the glucagon receptor suppresses the stimulatory effect of starvation on hepatic FGF21 mRNA abundance and plasma FGF21 concentration [19], [22]. To investigate whether glucagon acts directly on the liver to modulate hepatic FGF21 mRNA abundance and FGF21 secretion, experiments were carried out with primary cultures of rat hepatocytes incubated in serum-free medium containing or lacking glucagon. Incubating hepatocytes with glucagon stimulated a dose-dependent increase in FGF21 secretion into the culture medium (Fig. 1A). The stimulatory effect of glucagon on FGF21 secretion was maximal at 6 h of treatment (2.9-fold) (Fig. 1B). The glucagon-mediated increase in FGF21 secretion was reversible, as changing the culture medium from one containing glucagon to one lacking glucagon caused a marked decline in FGF21 secretion (Fig. 1C). Treatment with glucagon had no effect on albumin secretion into the culture medium. In contrast to the stimulatory effect of glucagon on FGF21 secretion, glucagon caused a transient decrease in FGF21 mRNA abundance that disappeared by 12 h of treatment (Fig. 1B). These findings demonstrate that glucagon acts directly on hepatocytes to induce FGF21 secretion and that a translational and/or posttranslational mechanism mediates this effect.

In addition to alterations in glucagon receptor signaling, changes in PPARα activity play a role in mediating the increase in hepatic FGF21 production and plasma FGF21 concentration during starvation [3], [4]. In contrast to the translational and/or posttranslational mechanism mediating the glucagon-induced increase on FGF21 secretion, PPARα activation enhances FGF21 secretion by increasing FGF21 gene transcription. The observation that glucagon receptor activation and PPARα activation modulate FGF21 secretion via distinct mechanisms led us to investigate the interaction between glucagon and the selective PPARα ligand/agonist GW7647 in the regulation of FGF21 secretion. Treatment of rat hepatocytes with glucagon plus GW7647 stimulated a greater increase in FGF21 secretion (14-fold) than treatment with glucagon (3-fold) or GW7647 (7.5-fold) alone (Fig. 1D). FGF21 mRNA levels in cells treated with glucagon plus GW7647 were lower than those observed in cells treated with GW7647 alone. The additive effect of glucagon and GW7647 on FGF21 secretion is consistent with the observation that glucagon and PPARα activation regulate FGF21 secretion by different mechanisms. This finding is also consistent with a role of both the glucagon receptor pathway and the PPARα pathway in mediating the increase in FGF21 production caused by starvation.

**Figure 2:**
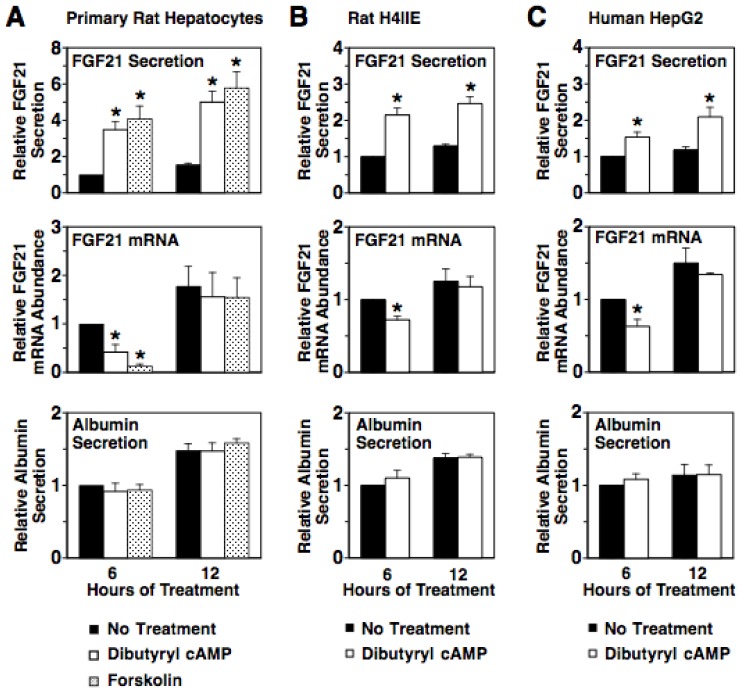
Dibutyryl cAMP and forskolin increase FGF21 secretion via a mechanism not involving changes in FGF21 mRNA abundance. Primary rat hepatocytes (A), rat H4IIE hepatoma cells (B) and human HepG2 hepatoma cells (C) were incubated with dibutyryl cAMP (100 µM) or forskolin (20 µM) for 6 and 12 h. The level of FGF21 and albumin in the culture medium and the abundance of FGF21 mRNA in total RNA of cells incubated with no treatments for 6 h were set at 1, and the other values were adjusted proportionately. Values are means ± SE (n = 4). * Significantly different (*P*<0.05) from that of cells incubated with no treatments.

Glucagon regulates metabolic processes by binding to a transmembrane spanning G protein-coupled receptor that activates adenylyl cyclase resulting in the increased production of the second messenger, cAMP [21]. This led us to investigate whether a membrane permeable form of cAMP (i.e. dibutyryl cAMP) or an adenylyl cyclase activator (i.e. forskolin) modulated FGF21 secretion. Incubating rat hepatocyte cultures with dibutyryl cAMP or forskolin stimulated a 3.5-4.1-fold increase in FGF21 secretion after 6 and 12 h of treatment (Fig. 2A). Dibutyryl cAMP was also effective in stimulating FGF21 secretion in the rat hepatoma cell line, H4IIE, and the human hepatoma cell line, HepG2 (Figs. 2B and 2C). Treatment with dibutyryl cAMP or forskolin had no effect on albumin secretion in rat hepatocytes, H4IIE cells, and HepG2 cells (Figs. 2A, 2B, and 2C). In contrast to the stimulatory effect of dibutyryl cAMP and forskolin on FGF21 secretion, these agents caused a transient decrease in FGF21 mRNA abundance that disappeared by 12 h of treatment (Figs. 2A, 2B, and 2C). Thus, agents that increase intracellular cAMP levels mimic the effects of glucagon on FGF21 secretion and FGF21 mRNA abundance. These observations confirm that the cAMP pathway induces hepatic FGF21 secretion and demonstrate that cAMP regulation of FGF21 secretion is retained in transformed cells and is conserved in humans.

### PKA and EPAC play a role in mediating the stimulatory effect of glucagon on FGF21 secretion

**Figure 3:**
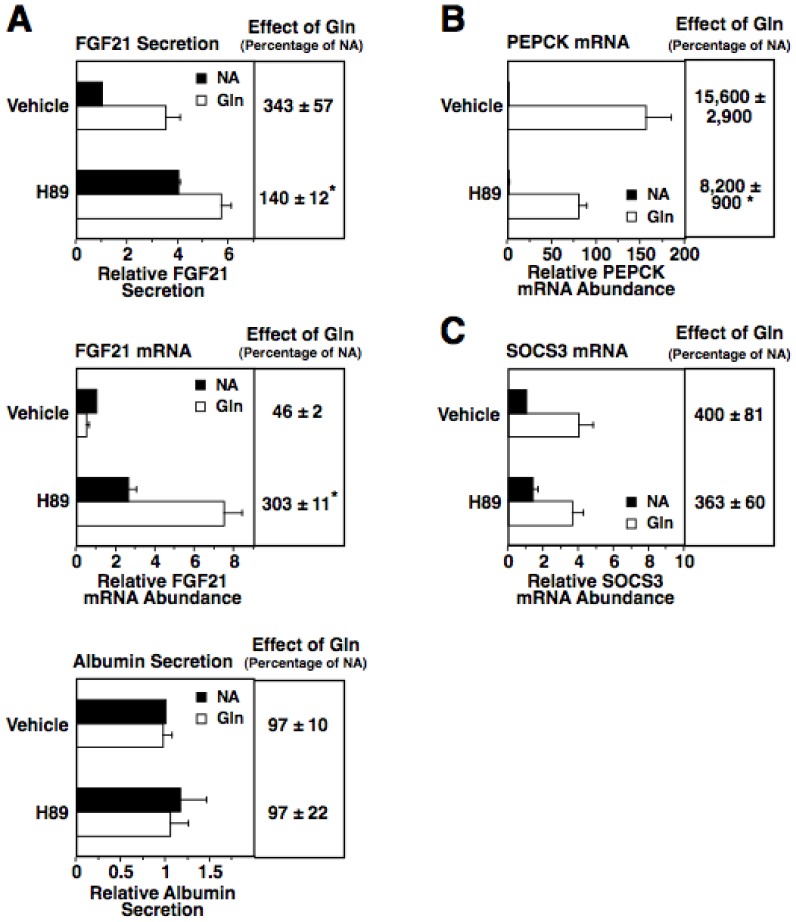
Inhibition of PKA suppresses the stimulatory effect of glucagon on FGF21 secretion. Primary rat hepatocytes were isolated and incubated in serum-free Medium 199. At 66 h of incubation, the medium was replaced with one of the same composition containing H89 (10 µM) or vehicle (DMSO) with or without glucagon, and the incubation was continued for 6 h. A: the level of FGF21 and albumin in the culture medium and the abundance of FGF21 mRNA in total RNA were measured. B: the abundance of PEPCK mRNA was measured. C: the abundance of SOCS3 mRNA was measured. Values for cells incubated with vehicle in the absence of glucagon were set at 1, and the other values were adjusted proportionately. The effect of glucagon is the level of FGF21 secretion, albumin secretion, FGF21 mRNA, PEPCK mRNA, or SOCS3 mRNA in cells treated with glucagon expressed as a percentage of the level in cells treated with NA. The effect of glucagon was calculated for individual experiments and then averaged. Values are means ± SE (n = 5). * Significantly different (*P*<0.05) from that of cells incubated with vehicle.

The glucagon-induced increase in intracellular cAMP concentration results in the activation of PKA [38]. We employed the PKA-specific inhibitor, H89, to investigate the role of PKA in mediating the effects of glucagon on FGF21 secretion. Incubating rat hepatocytes with H89 suppressed the stimulatory effect of glucagon on FGF21 secretion (Fig. 3A). Likewise, H89 suppressed the inhibitory effect of glucagon on FGF21 mRNA abundance. In fact, in presence of H89, FGF21 mRNA abundance was higher in cells incubated with glucagon compared to cells incubated without glucagon. H89 was also effective in suppressing the glucagon-induced expression of PEPCK (Fig. 3B), a previously identified PKA target gene [29]. In contrast, treatment with H89 had no effect on the glucagon-induced expression of SOCS3 (Fig. 3C), a gene that is regulated by glucagon solely via a PKA-independent mechanism [25], thus documenting the selectivity of H89. Treatment with H89 had no effect on albumin secretion in the absence or presence of glucagon. These findings provide support for a role of PKA in mediating the glucagon-induced increase in FGF21 secretion.

**Figure 4:**
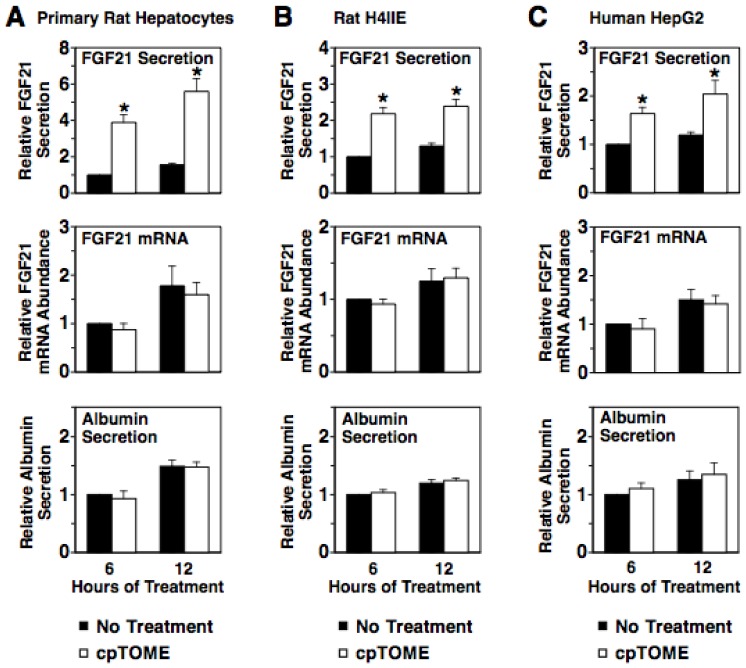
Activation of EPAC induces FGF21 secretion. Primary rat hepatocytes (A), rat H4IIE hepatoma cells (B) and human HepG2 hepatoma cells (C) were incubated with cpTOME (5 µM) for 6 and 12 h. The level of FGF21 and albumin in the culture medium and the abundance of FGF21 mRNA in total RNA of cells incubated with no treatments for 6 h were set at 1, and the other values were adjusted proportionately. Values are means ± SE (n = 4). * Significantly different (*P*<0.05) from that of cells incubated without cpTOME for the same time period.

In addition to stimulating PKA activity, the glucagon-induced increase in intracellular cAMP concentration activates EPAC, a guanine nucleotide exchange factor that activates the small GTPase Rap1 [31]. To investigate the role of EPAC in mediating the glucagon induction of FGF21 secretion, we first determined whether the EPAC-selective agonist, 8-(4-chlorophenylthio)-2'-O-methyladenosine- 3', 5'-cyclic monophosphate (cpTOME) [39], modulated FGF21 secretion. Incubating rat hepatocytes, H4IIE, or HepG2 cells with cpTOME for 6 and 12 h stimulated a 1.7-3.9-fold increase in FGF21 secretion but had no effect on FGF21 mRNA abundance and albumin secretion (Fig. 4). Thus, cpTOME mimics the translational and/or posttranslational effect of glucagon on FGF21 secretion.

**Figure 5:**
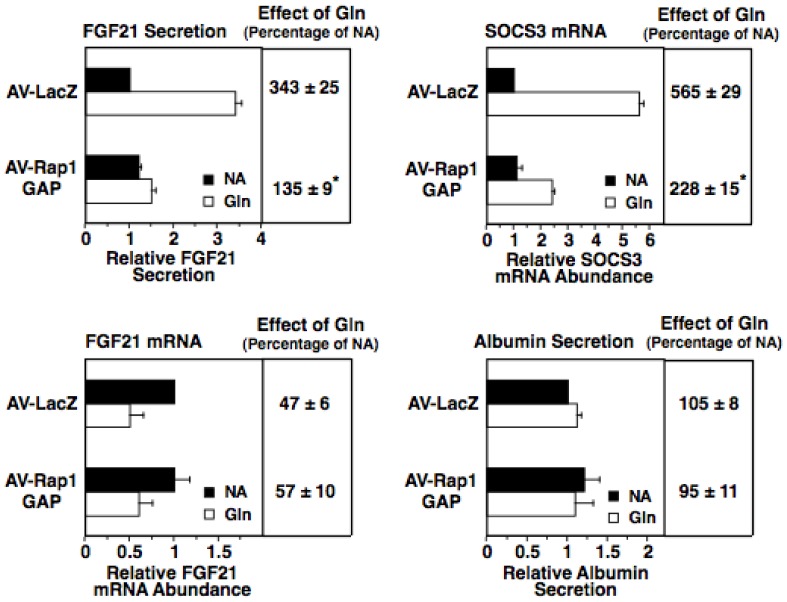
The glucagon-induced increase in FGF21 secretion is mediated by Rap1 activation. Primary rat hepatocytes were transduced with a Rap1GAP expressing adenovirus (AV-Rap1GAP) or a control adenovirus (AV-1LacZ). After 24 h, cells were incubated with or without glucagon for 24 h. The level of FGF21 and albumin in the culture medium and the abundance of FGF21 mRNA in total RNA of AV-LacZ-infected cells incubated with no additions (NA) were set at 1, and the other values were adjusted proportionately. The effect of glucagon was calculated as described in the legend of Fig. 3. Values are means ± SE (n = 4). * Significantly different (*P*<0.05) from that of cells infected with AV-LacZ.

Rap1 is a downstream mediator of EPAC action in hepatocytes [25]. To investigate the role of Rap1 in mediating the glucagon induction of FGF21 secretion, we determined the effect of ectopic expression of the Rap1 inhibitor, Rap1GAP, on FGF 21 secretion in rat hepatocytes. Rap1GAP inactivates Rap1 by accelerating GTP hydrolysis of activated Rap1 [40]. Expression of Rap1GAP from an adenoviral vector (AV-RapGAP) suppressed the ability of glucagon to increase FGF21 secretion (Fig. 5). Expression of Rap1GAP also suppressed the ability of glucagon to induce the previously identified EPAC/Rap1 target gene, SOCS3. In contrast, expression of Rap1GAP had no effect on the glucagon-mediated inhibition of FGF21 mRNA abundance. Albumin secretion was not altered by Rap1GAP expression in the absence or presence of glucagon. These results indicate that the EPAC/Rap1 pathway plays a role in mediating the glucagon induction of FGF21 secretion. In summary, the collective data from experiments employing inhibitors and/or activators of PKA and EPAC/Rap1 suggest that both of these branches of the cAMP pathway play a role in mediating the stimulatory effect of glucagon on FGF21 secretion.

### AMP-activated protein kinase (AMPK) and p38 MAPK are components of the glucagon/cAMP pathway controlling FGF21 secretion

**Figure 6:**
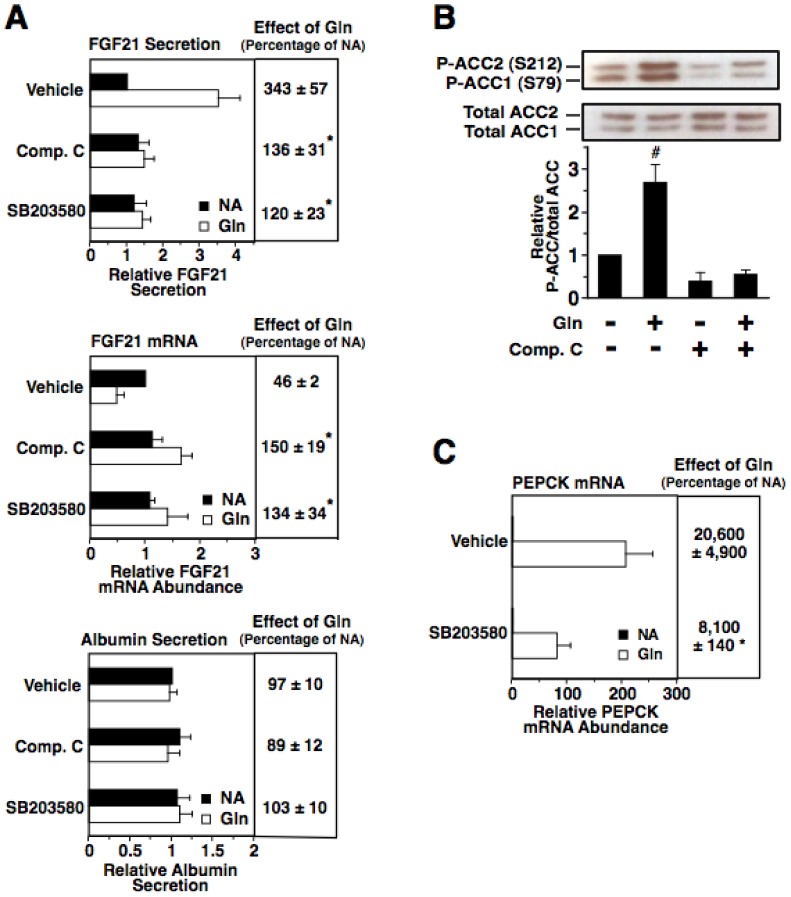
Inhibition of AMPK and p38 MAPK suppresses the stimulatory effect of glucagon on FGF21 secretion. Primary rat hepatocytes were isolated and incubated in serum-free Medium 199. At 66 h of incubation, the medium was replaced with one of the same composition containing compound C (5 µM), SB203580 (5 µM) or vehicle (DMSO) with or without glucagon, and the incubation was continued for 6 h. A: the level of FGF21 and albumin in the culture medium and the abundance of FGF21 mRNA in total RNA were measured. Values for cells incubated with vehicle in the absence of glucagon were set at 1, and the other values were adjusted proportionately. The effect of glucagon was calculated as described in the legend of Fig. 3. Values are means ± SE (n = 4). * Significantly different (*P*<0.05) from that of cells incubated with vehicle. B: the abundance of phosphorylated ACC1 (Ser^79^), phosphorylated ACC2 (Ser^212^), total ACC1, and total ACC2 in total cell lysates were measured by Western analysis. The ratio of phosphorylated ACC1 and ACC2 to total ACC1 and ACC2 of cells treated with no additions was set at 1, and the other values were adjusted proportionately. Values are means ± SE (n = 4). ^#^ Significantly different (*P*<0.05) from any other treatment group. C: the abundance of PEPCK mRNA in total RNA was measured.

Previous studies have shown that glucagon stimulates hepatic AMPK activity and that this effect is mediated, at least in part, by a PKA-dependent increase in the intracellular AMP/ATP ratio arising from the induction of gluconeogenesis [22], [41], [42], [43]. Glucagon may also increase AMPK activity via EPAC-dependent mechanism, as EPAC activation induces AMPK activity via a mechanism involving the activation of protein kinases (i.e. liver kinase B1 and calcium/calmodulin-dependent protein kinase kinase-β) that phosphorylate and activate AMPK [44]. To investigate the role of AMPK in mediating the glucagon-induced increase of FGF21 secretion, experiments were performed employing the selective and cell-permeable AMPK inhibitor, compound C [45]. Incubating rat hepatocytes with compound C suppressed the stimulatory effect of glucagon on FGF21 secretion (Fig. 6A). Compound C was also effective in suppressing the inhibitory effect of glucagon on FGF21 mRNA abundance. The effect of compound C on glucagon regulation of FGF21 secretion and FGF21 mRNA abundance was associated with a decrease in the glucagon-induced phosphorylation of the AMPK substrates, acetyl-CoA carboxylase 1 (ACC1) and acetyl-CoA carboxylase 2 (ACC2) (Fig. 6B). Treatment with compound C had no effect on albumin secretion in the absence or presence of glucagon (Fig. 6A). These findings provide support for a role of AMPK in mediating the stimulatory effect of glucagon on FGF21 secretion.

**Figure 7:**
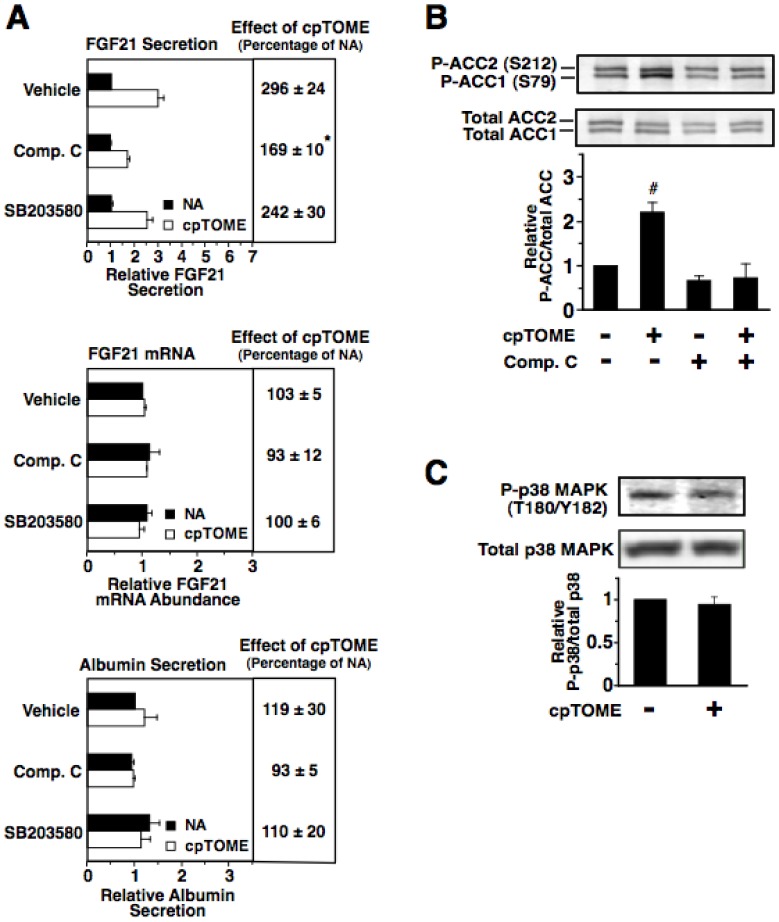
Inhibition of AMPK suppresses the stimulatory effect of EPAC activation on FGF21 secretion. Primary rat hepatocytes were isolated and incubated in serum-free Medium 199. At 66 h of incubation, the medium was replaced with one of the same composition containing compound C (5 µM), SB203580 (5 µM) or vehicle (DMSO) with or without cpTOME (5 µM), and the incubation was continued for 6 h. A: the level of FGF21 and albumin in the culture medium and the abundance of FGF21 mRNA in total RNA were measured. Values for cells incubated with vehicle in the absence of glucagon were set at 1, and the other values were adjusted proportionately. Values are means ± SE (n = 4). * Significantly different (*P*<0.05) from that of cells incubated with vehicle. B: the abundance of phosphorylated ACC1 (Ser^79^), phosphorylated ACC2 (Ser^212^), total ACC1, and total ACC2 in total cell lysates were measured by Western analysis. The ratio of phosphorylated ACC1 and ACC2 to total ACC1 and ACC2 of cells treated with no additions was set at 1, and the other values were adjusted proportionately. Values are means ± SE (n = 4). ^#^ Significantly different (*P*<0.05) from any other treatment group. C: the abundance of phosphorylated p38 MAPK (Thr^180^/Tyr^182^) and total p38 MAPK in cells treated with cpTOME for 6 h.

To investigate whether AMPK is linked to EPAC in the regulation of FGF21 secretion, we determined the effect of compound C on the induction of FGF21 secretion caused by cpTOME. Incubating rat hepatocytes with compound C suppressed the ability of cpTOME to increase FGF21 secretion (Fig. 7A). This effect was associated with a decrease in the cpTOME-induced phosphorylation of ACC1 and ACC2 (Fig. 7B). Treatment with compound C had no effect on FGF21 mRNA abundance and albumin secretion in the absence or presence of cpTOME (Fig. 7A). These observations provide support for the scenario that AMPK is a component of the EPAC/Rap1 pathway mediating the glucagon induction of FGF21 secretion.

Glucagon also increases the hepatic activity of p38 MAPK via a PKA-dependent mechanism [46], [47]. To investigate the role of p38 MAPK in mediating the glucagon-induced increase in FGF21 secretion, experiments were performed using the selective and cell-permeable p38 MAPK inhibitor, SB203580 [48]. Incubating rat hepatocytes with SB203580 suppressed the stimulatory effect of glucagon on FGF21 secretion and FGF21 mRNA abundance (Fig. 6A). Treatment with SB203580 was also effective in suppressing the glucagon-induced expression of the p38 MAPK target gene, PEPCK (Fig. 6C). Treatment with SB203580 had no effect on albumin secretion in the absence or presence of glucagon (Fig. 6A). These results provide support for a role of p38 MAPK in mediating the glucagon regulation of FGF21 secretion.

We also investigated the effect of SB203580 on the cpTOME regulation of FGF21 secretion. Treatment with SB203580 had no effect on the cpTOME-induced increase in FGF21 secretion (Fig. 7A). This finding suggests that p38 MAPK is not linked with EPAC/Rap1 in mediating glucagon regulation of FGF21 secretion. In support of this proposal, incubating rat hepatocytes with cpTOME had no effect on the amount of the active phosphorylated form (Thr180, Tyr182) of p38 MAPK (Fig. 7C). In summary, the collective findings suggest that both AMPK and p38 MAPK are components of the cAMP pathway mediating the glucagon-induced increase in FGF21 secretion. AMPK is a component of both the PKA branch and EPAC/Rap1 branch of the pathway, whereas p38 MAPK is a component of only the PKA branch of the pathway.

### Synergistic interaction between glucagon and insulin in the regulation of FGF21 secretion and FGF21 mRNA abundance

**Figure 8:**
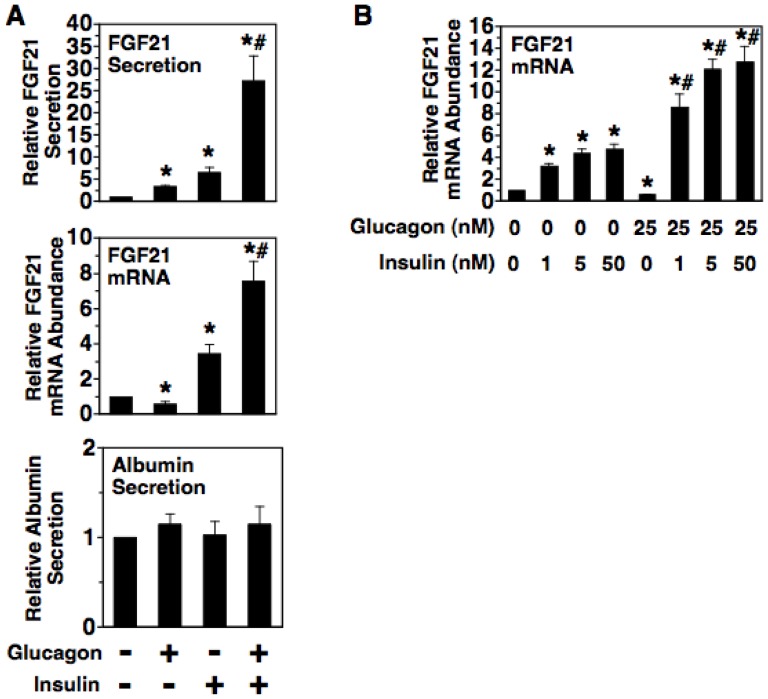
Glucagon synergistically interacts with insulin to induce FGF21 secretion and FGF21 mRNA abundance. A: effect of insulin on FGF21 secretion, FGF21 mRNA abundance, and albumin secretion in primary rat hepatocytes incubated in the absence and presence of glucagon. Cells were incubated with or without insulin (50 nM), glucagon (25 nM), or insulin plus glucagon for 6 h. The level of FGF21 and albumin in the culture medium and the abundance of FGF21 mRNA in total RNA of cells incubated no additions were set to 1, and the other values were adjusted proportionately. Values are means ± SE (n = 4). B: effect of different concentrations of insulin on FGF21 mRNA abundance. Hepatocytes were incubated with the indicated concentrations of insulin and glucagon for 6 h. The abundance of FGF21 mRNA in cells incubated with no hormones was set at 1, and the other values were adjusted proportionately. Values are means ± SE (n = 3). * Significantly different (*P*<0.05) from that of cells incubated with no additions. ^#^ Significantly different (*P*<0.05) from that of cells incubated with glucagon or insulin alone.

Previous studies performed in intact mice have shown that inhibition of the hepatic insulin signaling pathway by liver-specific ablation of insulin receptor substrate-1 and insulin receptor substrate-2 causes a decrease in hepatic FGF21 mRNA abundance during both the fed state and the starved state [49]. In addition, streptozotocin-induced diabetes in mice has been shown to suppress the stimulatory effect of starvation on hepatic FGF21 mRNA abundance [50]. Although insulin is generally regarded as a hormone signaling the fed state, these observations suggest that insulin plays a role in mediating the increase in FGF21 mRNA abundance during the starved state. We investigated this possibility by determining the effects of insulin on FGF21 mRNA abundance and FGF21 secretion in rat hepatocyte cultures incubated with or without the starvation signal glucagon. Incubating rat hepatocytes with insulin alone for 6 h stimulated a 7-fold increase in FGF21 secretion (Fig. 8A). This effect was associated with a 3.5-fold increase in FGF21 mRNA abundance. Incubating hepatocytes with insulin plus glucagon stimulated a substantially greater increase in FGF21 secretion (28-fold) and FGF21 mRNA abundance (7.6-fold) relative to that observed in cells incubated with insulin or glucagon alone. Treatment with insulin in the absence or presence of glucagon had no effect on albumin secretion. Dose-response experiments demonstrated that insulin was effective in stimulating FGF21 mRNA abundance at a concentration observed in the portal vein during fasted conditions (i.e. 1 nM) [51] (Fig. 8B). These findings demonstrate that insulin at physiological concentrations causes an increase in FGF21 secretion and that alterations in FGF21 mRNA abundance play a role in mediating this response. These findings also identify a novel synergistic interaction between insulin and glucagon in the regulation of FGF21 secretion and FGF21 mRNA abundance. Interestingly, the presence of insulin unmasks the ability of glucagon to increase in FGF21 mRNA abundance. Overall, the results of these experiments performed in primary hepatocyte cultures provide support for the aforementioned in vivo studies [49], [50] demonstrating that the insulin signaling pathway enhances hepatic FGF21 gene expression during both fed and starved conditions.

Habegger et al. [52] and Uebanso et al. [53] have recently reported that glucagon treatment of mouse hepatocyte cultures stimulates a 1.6-2.2-fold increase in FGF21 mRNA abundance or FGF21 gene transcription. In these studies, hepatocytes were initially plated in medium containing insulin and 10% fetal bovine serum. It was unclear whether subsequent incubations were performed in medium containing or lacking insulin. If insulin was present in the incubation medium, this would explain why Habegger and Uebanso observed a stimulatory effect of glucagon on FGF21 mRNA abundance rather than a transient inhibitory effect of glucagon on FGF21 mRNA abundance as reported here for rat hepatocytes incubated in the absence of insulin.

## Discussion

The glucagon receptor pathway plays a key role in mediating the stimulatory effect of starvation on FGF21 production [22]. In the present study, we show that glucagon acts directly on hepatocytes to increase FGF21 secretion and that the mechanism mediating this effect does not involve changes in FGF21 mRNA abundance. To our knowledge, this is the first evidence that a starvation signal modulates FGF21 secretion via a translational and/or posttranslational mechanism. Another signaling pathway contributing to the starvation-induced increase in FGF21 production is PPARα [3], [4], [5]. PPARα activation increases FGF21 expression by stimulating the transcription of the FGF21 gene. Collectively, these observations suggest that both transcriptional and posttranscriptional processes play a role in mediating the elevation in hepatic FGF21 production caused by starvation.

While the mechanisms controlling FGF21 gene transcription are well described [54], little is known about the posttranscriptional mechanisms controlling FGF21 expression and secretion. Previous studies have shown that posttranscriptional mechanisms play a significant role in regulating the secretion of other hepatic proteins. For example, insulin inhibits apolipoprotein B (apoB) secretion in hepatocytes by decreasing the translation of apoB mRNA and by increasing the degradation of apoB protein [55], [56]. The stimulatory effect of high-fat and high-cholesterol consumption on hepatic apolipoprotein A-1 (apoA-1) secretion is mediated by an increase in the translation of apoA-1 mRNA [57]. Future studies will investigate the role of FGF21 mRNA translation and FGF21 protein degradation in mediating the effect of glucagon on FGF21 secretion.

**Figure 9:**
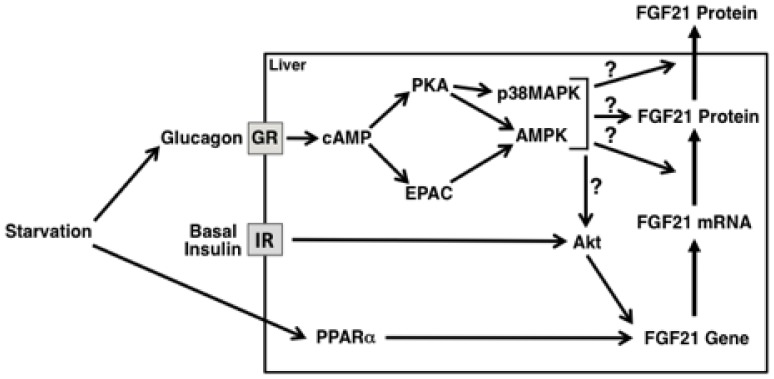
Proposed model for how starvation increases hepatic FGF21 gene expression and secretion. Starvation increases the secretion of glucagon into the portal circulation. At the liver, glucagon binds to the glucagon receptor (GR) triggering a signaling cascade resulting in the activation of the PKA and EPAC branches of the cAMP pathway. Activation of PKA and EPAC stimulates FGF21 secretion via a translational and/or posttranslational mechanism. Additional components of the glucagon pathway regulating FGF21 secretion include P38 MAPK and AMPK; these proteins function downstream of PKA and/or EPAC. Glucagon also increases FGF21 secretion via a pretranslational mechanism, and this effect is unmasked by the presence of insulin. Activation of Akt may play a role in mediating the effect of glucagon and insulin on FGF21 gene expression. The activation of PPARα also contributes to the starvation-induced increase in FGF21 gene expression. IR: insulin receptor.

The results of the present study also delineate the signaling pathways through which glucagon regulates FGF21 secretion. Glucagon increases FGF21 secretion through both the PKA branch and the EPAC branch of the cAMP pathway. AMPK and p38 MAPK are components of one or both of these pathways controlling FGF21 secretion (Figs. 2–7 and 9). Previous studies have shown that both PKA and EPAC play a role in mediating the effects of cAMP on other cellular processes. For example, the stimulatory effect of cAMP on glucose-induced insulin secretion in pancreatic beta cells and the inhibitory effect of glucagon on bile acid-induced apoptosis in hepatocytes are dependent on the presence of both PKA and EPAC [58], [59]. This dual regulation by PKA and EPAC may expand the dynamic range of cAMP signaling, as the activation of PKA-mediated events is proposed to occur at lower cAMP levels than the activation of EPAC-mediated events [60].

Habegger and coworkers [52] have shown that chronic administration of a synthetic glucagon receptor agonist to obese mice causes a reduction in body weight and body fat content via a FGF21-dependent mechanism. The physiological actions of glucagon in liver may also be mediated, at least in part, by a FGF21-dependent mechanism, as glucagon and FGF21 exert similar effects on hepatic fatty acid oxidation and gluconeogenesis [2], [19]. Glucagon activation of fatty acid oxidation and gluconeogenesis requires the presence of AMPK and/or p38 MAPK [43], [46], [61]. Interestingly, our data indicate that glucagon induction of FGF21 secretion also requires the presence of AMPK and p38 MAPK (Fig. 6). These observations provide support for the hypothesis that FGF21 functions as a mediator of glucagon action.

Our results suggest that AMPK is a component of both the PKA pathway and the EPAC pathway mediating the cAMP regulation of FGF21 secretion (Figs. 6, 7, and 9). The interconnection of the PKA pathway with the EPAC pathway through a common downstream protein has been described for other cAMP responsive processes. For example, the PKA-dependent and the EPAC-dependent effects of cAMP on glucose-induced insulin secretion in beta cells are mediated through the exocytosis regulatory protein, snapin [62]. PKA phosphorylates snapin at serine 50, stimulating an increase in the formation of an EPAC2-containing protein complex that promotes insulin exocytosis. Other studies have shown that cAMP regulation of cardiomyocyte hypertrophy is mediated by the interaction of PKA and EPAC with a protein complex comprised of muscle-specific A-kinase anchoring protein, phosphodiesterase 4DE, and extracellular signal-regulated kinase 5 [63]. These observations in combination with the present findings analyzing the cAMP regulation of FGF21 secretion suggest that interaction of PKA and EPAC with a common downstream protein is a general mechanism of cAMP signaling.

Another key finding of the present study is that insulin increases FGF21 secretion and FGF21 mRNA abundance and interacts with glucagon in a synergistic manner to induce a further increase in FGF21 secretion and FGF21 expression (Fig. 8). The synergistic interaction between insulin and glucagon in the regulation of FGF21 expression is significant for several reasons. First, it provides an explanation for why administration of glucagon or a synthetic glucagon receptor agonist to normal intact mice causes an increase in not only plasma FGF21 concentration but also hepatic FGF21 mRNA abundance [22], [52]. Second, this phenomenon provides a mechanism accounting for the increase in plasma FGF21 concentration and hepatic FGF21 mRNA abundance observed in animals and patients with type 2 diabetes [64], [65], a condition that is associated with an elevation in the plasma concentration of both glucagon and insulin. Third, this phenomenon provides an explanation for why glucagon receptor ablation in mice attenuates the stimulatory effect of starvation on hepatic FGF21 mRNA abundance [22]. During the normal 24 h feeding cycle in rats, the portal insulin concentration varies by approximately 1.2-fold, whereas the portal glucagon concentration varies by a significantly greater extent (i.e. 1.6-fold) [51]. We postulate that insulin maintains a basal level of hepatic FGF21 mRNA abundance during the carbohydrate-fed state and synergistically interacts with elevated glucagon receptor signaling during the starved state to induce a further increase in hepatic FGF21 mRNA abundance (Fig. 9). This proposal is supported by in vivo studies demonstrating that liver-specific disruption of the insulin signaling pathway causes a decrease in hepatic FGF21 mRNA abundance during both carbohydrate-fed and starved conditions and that streptozotocin-induced diabetes suppresses the ability of starvation to increase hepatic FGF21 mRNA abundance [49], [50].

Given that glucagon opposes the effects of insulin on a wide variety of metabolic processes, it is somewhat surprising that glucagon enhances the ability of insulin to stimulate FGF21 expression. However, glucagon has been shown to interact cooperatively with insulin in regulating other hepatic processes. For example, glucagon potentiates the ability of insulin to stimulate hepatic DNA synthesis and cell proliferation [66], [67], [68]. Other studies have shown that glucagon enhances the suppressive effect of insulin on hepatic glucose production under certain experimental conditions [69], [70]. A regulatory mechanism involving a cooperative interaction between insulin and glucagon may have evolved to allow for the induction of FGF21 gene expression during a broad range of stress conditions including starvation and diseases arising from overnutrition (e.g. type 2 diabetes).

What is the mechanism mediating the synergistic interaction of glucagon and insulin in the regulation of FGF21 mRNA? Previous studies have shown that glucagon inhibits insulin signal transduction at a step downstream of mammalian target of rapamycin complex 1 (mTORC1) [71]. Thus, the glucagon/insulin synergistic interaction is likely mediated by a step upstream of mTORC1. Interestingly, it has been reported that glucagon enhances the ability of insulin to stimulate Akt activity in rat hepatocytes [72], an observation confirmed by our laboratory (S. Bhatnagar and F.B. Hillgartner, unpublished observation). Other work has shown that muscle-specific overexpression of Akt1 increases muscle FGF21 mRNA abundance in mice [73]. Future studies will investigate the role of Akt in mediating the synergistic interaction of glucagon and insulin in the regulation of FGF21 mRNA abundance.

In conclusion, glucagon induces hepatic FGF21 secretion by a translational and/or posttranslational mechanism (Fig. 9). This effect is mediated by the activation of the PKA branch and the EPAC branch of the cAMP pathway and requires the presence of AMPK and p38 MAPK. Glucagon also increases FGF21 secretion by a pretranslational mechanism, and this effect requires the presence of insulin. We propose that glucagon acts directly on the liver in mediating the starvation-induced increase in hepatic FGF21 secretion and FGF21 mRNA abundance and that the presence of insulin facilitates this response. The activation of PPARα also contributes to the starvation-induced increase in hepatic FGF21 secretion and FGF21 mRNA abundance.
